# Event and Comment

**Published:** 1919-07

**Authors:** 


### DENTAL REGISTER

THE MAGAZINE OF DENTISTRY

Vol. LXXIII JULY, 1919 No. 7

### EVENT AND COMMENT

**Artistic Prosthesis** We are reprinting in this issue an article written by Dr. J. Leon Williams, which appears in the June issue of *The Dental Digest* as a comment on a paper by Dr. F. H. Orton of St. Paul, which was published in *The Dental Summary* of April, 1919, both of which we would earnestly commend to the careful consideration of all dentists who aim to serve their patients to the ideal degree. Dr. Williams' definition of art as it is to be developed in our calling is both ideal and practical. In fact, he assures us that only the ideal can be practical, at least he makes his comment so specific that he leaves us no other logical conclusion. We may not all have this conception of our prosthetic endeavors, as he very truly says, and most of us, perhaps, can have little sympathy with so high a conception as he apparently has in mind as he writes, but it seems to us he has issued a challenge to us that we should not let pass without the most careful consideration.

If we take up only one point of his address—that of color harmony in dental prosthesis—and examine ourselves thoughtfully, we shall soon find that in practical endeavors our methods in the past have been confined entirely to matching tooth colors, as we superficially observe them; or, as our circumstances enable us to produce effects which do not shock our patients by their gross inharmonies. Dr. Orton in his paper blames the tooth manufacturer for much of the failure of dental prosthetists to produce a higher type of color harmony, while Dr. Williams says very properly this is not the whole trouble. Dr. Williams asserts further, that when the profession knows what is wanted that the manufacturers will supply the demand. We are not at all sure of this, for we have an idea that it will be about as impracticable for porcelain tooth manufacturers to make truly artistic porcelain teeth in the desired quantities to meet the needs of all classes of people, as it is for present-day methods to produce artistic pottery in sufficient quantity to meet the needs of domestic service in table dishes that are beautiful enough to stimulate the appetite for ordinary food. The manufacturers of artistic vases are delighted if they can secure one real prize piece of pottery in hundreds of strenuous well-directed endeavors. This, of course, is due to the practicable impossibility of accurately laying on the colors and applying the heat treatment with such precision that any predetermined result may be assured. Now, since porcelain teeth are produced by much the same processes, we can never expect much better results as long as teeth are produced in such quantities and by present methods. Undoubtedly, we shall improve our tooth colors in time, just as we have already done with tooth forms and colors from the original colors and forms as first produced by Fonzi and others, and we should not cease to strive for a better or more ideal product, especially if there is any possibility or hope of attaining so desired a result.

We also believe that there is gross neglect as well as much ignorance prevalent in the matter of selecting and adapting the teeth which are now available. All one needs to do to convince himself of this fact is that he attend some social reception, where a large number of older persons appear in dress parade, and give especial attention to the shocking inharmonies in the dental toilets as compared with other environments. The wonder is that people of refined inclinations will tolerate the incongruities generally seen in such places. In many cases nothing in the way of artistic replacement seems to have been considered, either in materials or mchanical construction or adaptation. A consideration of this line of thought would lead us too far away, however, from the purpose of this comment, as it is a subject which at no distant day will surely have to be considered more thoughtfully than it has ever been. Let us hope it may be studied both artistically and scientifically in the sense of Dr. Williams' definition of what true art really is or should be.

We have studied the mechanics of dental art to good purpose but we shall never gain any true conceptions of the ideal until we get a better conception of what nature is or should be in the typical. Even then, the natural will not artistically represent the harmonious. In the wonderful work of Dr. D. D. Smith in creating hygienic oral conditions by polishing the teeth at frequent intervals, he discovered that there were only a few shades of color in the natural teeth, and that there was not so much variation in different ages of the patients as we had formerly supposed. It should be possible to standardize our porcelain, and possibly to so glaze it that we should get a more artistic refraction of light and then more beautiful and artistic color results, and it should be accomplished with such simple combinations that even the thoughtless dentists could not make such manifest blunders as are all too common today.

